# ABHD18 degrades cardiolipin by stepwise hydrolysis of fatty acids

**DOI:** 10.1016/j.jbc.2025.110237

**Published:** 2025-05-14

**Authors:** Mindong Ren, Shiyu Chen, Miriam L. Greenberg, Michael Schlame

**Affiliations:** 1 Department of Anesthesiology, New York University Grossman School of Medicine, New York, New York, USA; 2 Department of Biological Sciences, Wayne State University, Detroit, Michigan, USA

**Keywords:** cardiolipin, lipase, lysophospholipid, mitochondria, phospholipid turnover, tafazzin

## Abstract

Cardiolipin (CL), the signature phospholipid of mitochondria, carries four fatty acids that are remodeled after *de novo* synthesis. In yeast, remodeling is accomplished by the joint action of Cld1, a lipase that removes a fatty acid from CL, and Taz1, a transacylase that transfers a fatty acid from another phospholipid to monolysocardiolipin (MLCL). While *taz1* homologs have been identified in all eukaryotes, *cld1* homologs have remained obscure. Here, we demonstrate that α/β-hydrolase domain 18 (ABHD18), a highly conserved protein of plants, animals, and humans, is functionally homologous to Cld1. Knockdown of *Abhd18* decreased the concentration of MLCL in murine, *Taz*-knockout myoblasts. Inactivation of *Abhd18* in *Drosophila* substantially increased the abundance of CL. *Abhd18* inactivation also reversed the increase in the rate of CL degradation, as measured with ^13^C isotopes, and the accumulation of deacylated CLs, such as MLCL and dilyso-CL, in tafazzin (TAZ)-deficient flies. CL species with more than five double bonds were resistant to ABHD18. Our data demonstrate that ABHD18 is the elusive lipase that hydrolyzes CL in mice and flies and presumably in other organisms. Rather than removing just one fatty acid, we show that ABHD18 deacylates CL further. Thus, ABHD18 catalyzes the breakdown of CL, whereas TAZ protects CL from degradation.

Cardiolipin (CL) is a unique glycerophospholipid with four acyl chains. In eukaryotes, it is found predominantly in the inner mitochondrial membrane, where it plays a crucial role in bioenergetic function (2, 3, 4). After *de novo* synthesis, CL undergoes a remodeling process to achieve its mature form, which is rich in unsaturated fatty acids. This remodeling is conserved in evolution and requires repeated cycles of CL deacylation and reacylation to remodel all four chains (5). The acyl transferase tafazzin (TAZ) plays a critical role in CL remodeling as it can convert monolysocardiolipin (MLCL) into CL and *vice versa* (6). Mutations in the *Taz* gene are associated with Barth syndrome, a rare X-linked multisystemic disorder characterized by CL deficiency, MLCL accumulation, and abnormal CL species (7). In yeast, the deacylation step of CL remodeling is catalyzed by the lipase Cld1 (8). In TAZ-deficient yeast, deletion of *cld1* not only suppresses MLCL accumulation and restores CL content but also rescues its growth and lifespan defects, suggesting that the inhibition of the human homolog of *cld1* is a potential strategy to treat Barth syndrome (9, 10). However, no *cld1* homolog has been identified in humans up to now.

The protein sequence of Cld1 contains a putative N-terminal signal for mitochondrial targeting and an α/β-hydrolase domain (ABHD). To identify Cld1 candidates in higher eukaryotes, we decided to focus on ABHD proteins. Among the 23 mammalian ABHD proteins annotated in UniProt, ABHD4 is most closely related to Cld1 by phylogenetic analysis (11). However, it does not contain a mitochondrial targeting sequence. In contrast, ABHD10, ABHD11, and ABHD18 have been demonstrated experimentally to reside in mitochondria (12, 13, 14) and only they are predicted by the latest deep learning protein localization programs (DeepLoc and MULocDeep) to contain N-terminal mitochondrial targeting sequences (Table S1). Unlike ABHD10 and ABHD11 whose functions have been well characterized, ABHD18 is a mitochondrial protein of unknown function. In AlphaFold, the catalytic triad (Ser-Asp-His) of Cld1 (15) is nearly superimposable with that of ABHD18, and both enzymes can accommodate CL in their substrate binding pockets (Fig. S1). In this article, we searched for the homolog of *cld1* in higher eukaryotes and found that *Abhd18* replicates all effects of *cld1* on CL metabolism.

## Results

**Figure 1:**
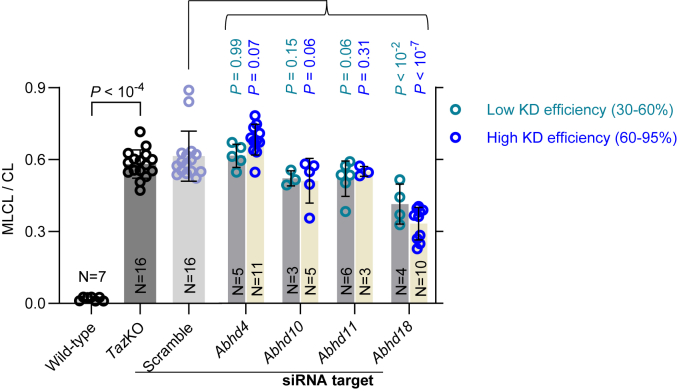
**Knockdown (KD) of ABHD18 reduces the MLCL/CL ratio in *Taz*KO myoblasts.** ABHDs were knocked down by different siRNAs in C2C12 myoblasts carrying *Taz*KO. Scrambled (Src) siRNA served as control. KD efficiencies were measured by quantitative RT–PCR, and MLCL/CL ratios were measured by mass spectrometry. Graphs show mean values with standard deviations of multiple biological replicates. Comparisons were made by Student's *t* test. ABHD, α/β-hydrolase domain; CL, cardiolipin; MLCL, monolysocardiolipin.

To identify the mammalian homolog of *cld1*, we silenced the messages of candidate *Abhd*s, including *Abhd4*, *Abhd10*, *Abhd11*, and *Abhd18*, in C2C12 myoblasts that carried a knockout in the *Taz* gene (*Taz*KO). Because *cld1* deletion normalizes the MLCL/CL ratio in *Taz*KO yeast (8, 9), we expected that knockdown of its mammalian homolog will reduce the MLCL/CL ratio in *Taz*KO myoblasts. Among the four genes, only knockdown of *Abhd18* produced a statistically significant decrease in the MLCL/CL ratio (Fig. 1). These data tentatively suggest that *Abhd18* is the *cld1* homolog.

**Figure 2:**
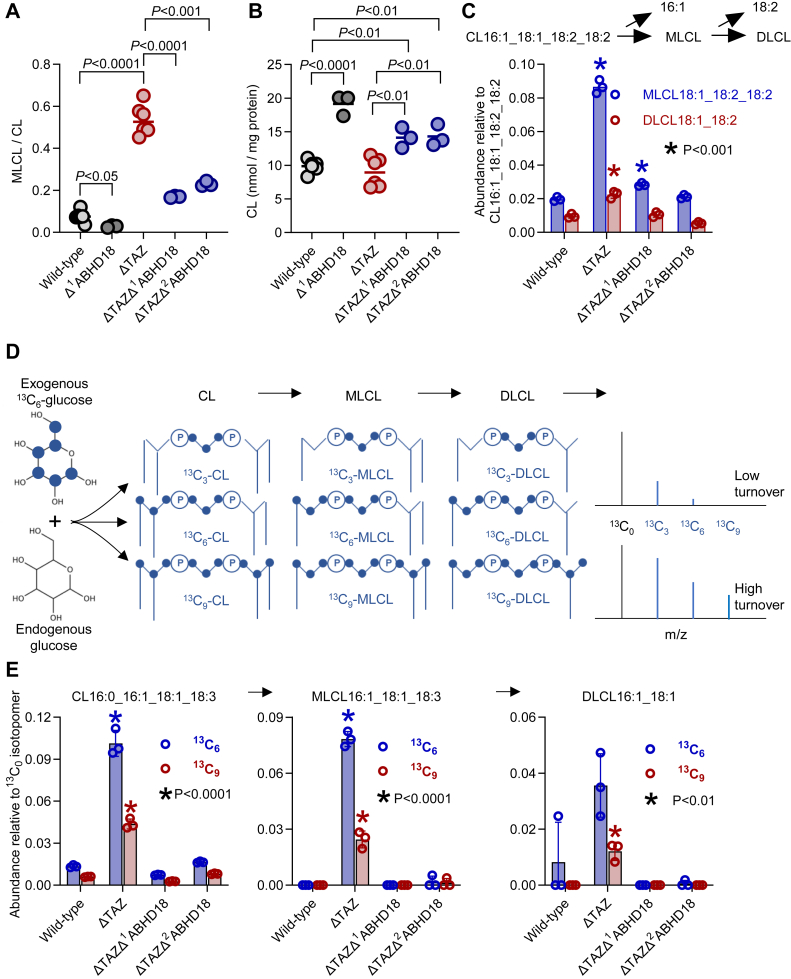
**Inactivation of *Abhd18* inhibits CL hydrolysis in ΔTAZ *Drosophila*.***A* and *B*, CL and MLCL were measured in lipid extracts of whole flies by mass spectrometry. *C*, specific hydrolysis products of CL16:1_18:1_18:2_18:2 were measured in lipid extracts of fly thoraces by mass spectrometry. *D*, when flies are incubated with ^13^C_6_-glucose, metabolic turnover of CL produces isotopomers with one, two, or three ^13^C_3_-glycerols in CL and its degradation products (MLCL and DLCL). The faster the turnover the higher are the relative signal intensities of ^!3^C_3_, ^13^C_6_, and ^13^C_9_ isotopomers. *E*, flies were fed ^13^C_6_-glucose for 2 days, thoraces were excised, and lipids were analyzed by mass spectrometry. Abundances of ^13^C_6_ and ^13^C_9_ isotopomers of the indicated species were divided by the abundance of the ^13^C_0_ isotopomer. Graphs show mean values with standard deviations of biological replicates. *Asterisks* indicate a statistically significant difference to the corresponding measurement in wildtype. Comparisons were made by Student's *t* test. ABHD, α/β-hydrolase domain; CL, cardiolipin; DLCL, dilysocardiolipin; MLCL, monolysocardiolipin; TAZ, tafazzin.

To directly test this hypothesis, we inactivated *Abhd18* in *Drosophila* carrying the *Taz* deletion (ΔTAZ). This was accomplished by crossing ΔTAZ flies with two commercial fly strains that contained separate transposon insertions in *Abhd18*. The inserts reduced expression of the *Abhd18* message by 100 ± 0% (Δ^1^ABHD18, N = 3) and 96 ± 1% (Δ^2^ABHD18, N = 3) respectively, without producing an obvious phenotype, except for a very small but statistically significant decrease in the MLCL/CL ratio. However, in the ΔTAZ background, *Abhd18* inactivation rescued male sterility (from 0 ± 0% fertility in ΔTAZ to 98 ± 1% fertility in ΔTAZΔ^1^ABHD18 and 97 ± 30% fertility in ΔTAZΔ^2^ABHD18, N = 3) and strongly reduced the MLCL/CL ratio to near wildtype levels (Fig. 2*A*).

The data identify *Abhd18* as the *Drosophila* homolog of *cld1*. *Abhd18* inactivation did not only reversed the elevation of MLCL in ΔTAZ but also increased the concentration of CL above wildtype levels (Fig. 2*B*). The decrease in MLCL could not account for the substantial increase in CL, raising the question whether ABHD18 catalyzes further deacylations. Indeed, *Abhd18* inactivation reduced the abundance of a few detectable dilysocardiolipin (DLCL) species. For instance, it reduced the abundance of DLCL18:1_18:2 in addition to that of its precursor MLCL18:1_18:2_18:2 (Fig. 2*C*).

The data indicate that ABHD18 catalyzes not only the first but also the second deacylation of CL, raising the prospect of subsequent deacylations that may lead to complete degradation. To determine whether ABHD18 causes CL degradation, we incubated flies with ^13^C_6_-glucose. If CL is actively synthesized and degraded, ^13^C is incorporated primarily into glycerol moieties (16). This gives rise to a series of isotopomers with either three, six, or nine heavy carbons in CL and partially degraded CLs (Fig. 2*D*). We have shown before that the relative abundance of ^13^C_3_, ^13^C_6_, and ^13^C_9_ isotopomers of CL species measures their metabolic turnover in postmitotic tissues like *Drosophila* flight muscles (16, 17). We confirmed that the turnover of CL was extremely slow in wildtype thoraces (flight muscles) but drastically increased in ΔTAZ along with the turnover of MLCL and DLCL. For example, deletion of *Taz* increased turnover of CL16:0_16:1_18:1_18:3 and that of its lysophospholipids, directly proving an elevated CL degradation in ΔTAZ. Inactivation of *Abhd18* fully reversed the effect of *Taz* deletion on the turnover of CL, MLCL, and DLCL, which corroborates that ABHD18 breaks down CL by repeated deacylations (Fig. 2*E*).

**Figure 3:**
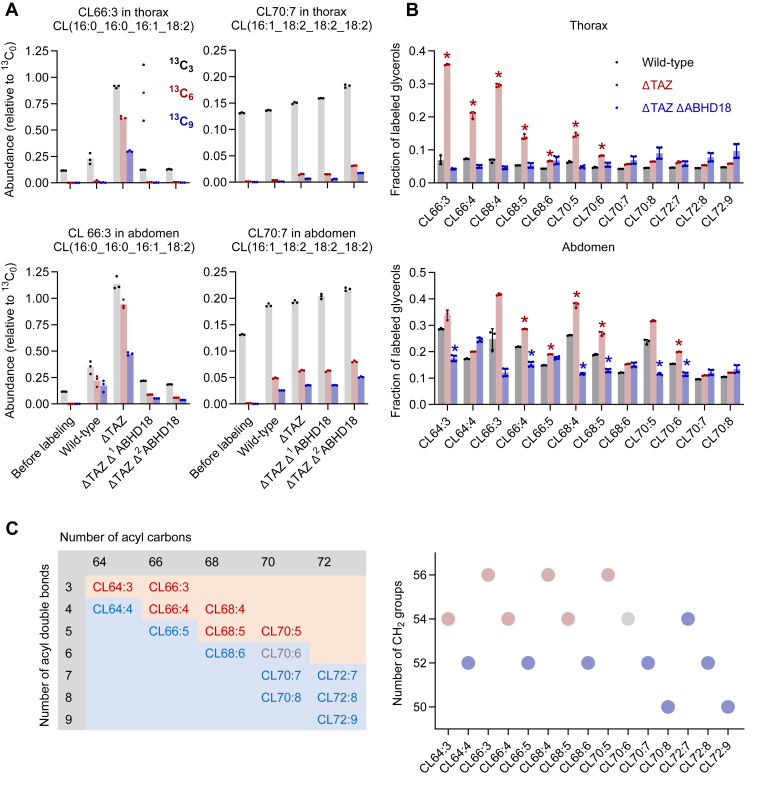
**Molecular species of CL are not equally susceptible to ABHD18.** Flies were fed ^13^C_6_-glucose for 2 days. Lipid extracts from thoraces and abdomens were analyzed by mass spectrometry. Bar graphs show mean values with standard deviations of biological replicates. *A*, abundances of ^13^C_3_, ^13^C_6_, and ^13^C_9_ isotopomers of the indicated species were divided by the abundance of the ^13^C_0_ isotopomer. Ratios were higher in abdomens than in thoraces because abdominal tissues regenerate faster than thoracic tissues. *B*, proportions of ^13^C-labeled glycerol were calculated from isotopomer patterns. *Asterisks* indicate a statistically significant difference to wildtype (*p* < 0.0001, Student's *t* test). *C*, molecular species of CL from thorax and abdomen are arranged according to chain length and unsaturation. The plot shows the number of methylene groups that each species carries in the acyl chains. CL species shown in *red* decreased their turnover in response to *Abhd18* inactivation. CL species shown in *blue* were not affected by *Abhd18* inactivation. ABHD, α/β-hydrolase domain; CL, cardiolipin.

Surprisingly, not all molecular species of CL were susceptible to ABHD18 *in vivo*. For instance, the turnover of CL16:0_16:0_16:1_18:2 increased upon *Taz* deletion and decreased upon *Abhd18* inactivation, but the turnover of CL16:1_18:2_18:2_18:2 hardly changed upon *Taz* deletion and slightly increased upon *Abhd18* inactivation (Fig. 3*A*). The same trend was observed when we analyzed *Drosophila* abdomens instead of thoraces. Among 15 CL species included in our analysis, six species (CL64:3, CL66:3, CL66:4, CL68:4, CL68:5, and CL70:5) were strongly affected by TAZ and ABHD18. The turnover rates of other species remained largely unchanged by the deletions (Fig. 3*B*). CL species that were susceptible to ABHD18 carried more acyl carbons and less double bonds than species that were not susceptible. In essence, susceptibility correlated with the number of methylene groups in the acyl chains of CL (Fig. 3*C*).

**Figure 4:**
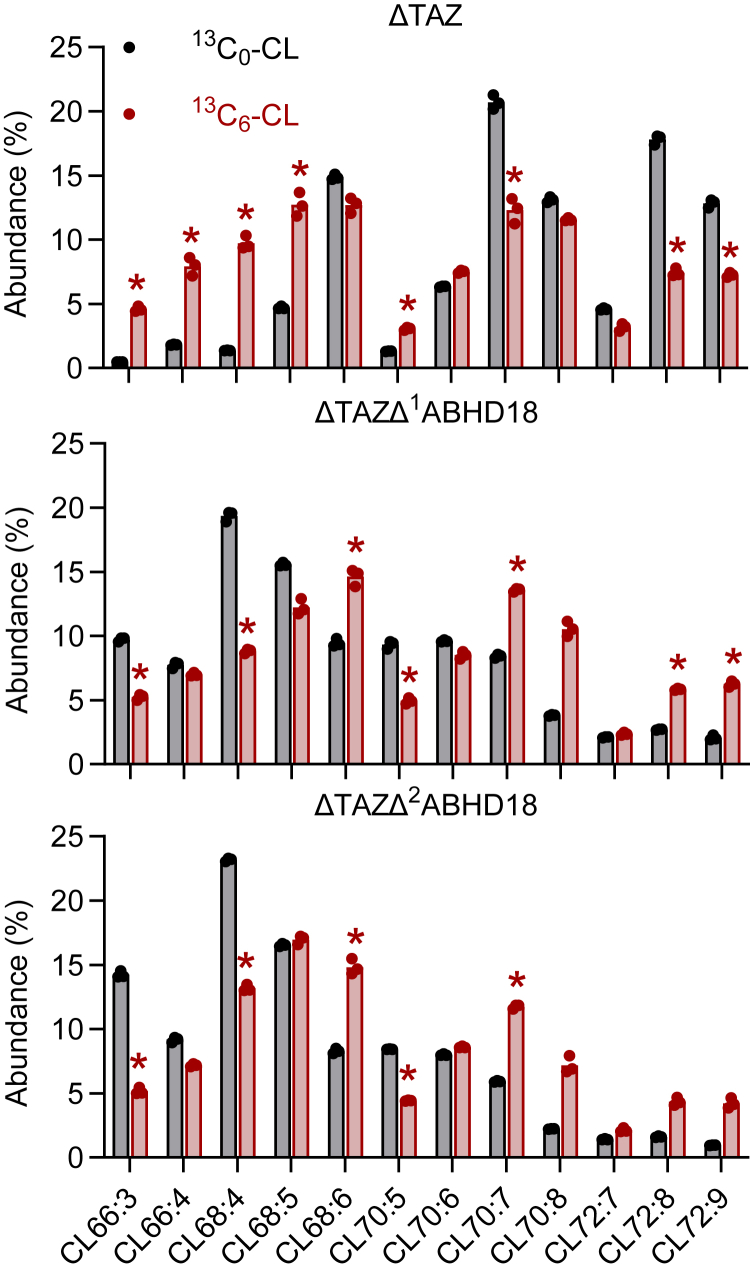
**ABHD18 modifies the molecular species composition of CL.** Flies were fed ^13^C_6_-glucose for 2 days. Thoraces were isolated, and lipids were analyzed by mass spectrometry. Molecular species patterns were calculated from the signal intensities of ^13^C_0_ isotopomers and ^13^C_6_ isotopomers, respectively. Bar graphs show mean values (N = 3) of biological replicates. *Asterisks* indicate a statistically significant difference between ^13^C_0_-CL and ^13^C_6_-CL (*p* < 0.001, Student's *t* test). ABHD, α/β-hydrolase domain; CL, cardiolipin.

To determine whether species-selective degradation of CL influences the molecular composition, we compared newly synthesized (^13^C-labeled) CL with pre-existing (unlabeled) CL in ΔTAZ flies. We reasoned that ^13^C-labeled CL is mostly shaped by synthesis, whereas unlabeled CL is shaped by both synthesis and degradation. Following 2 days of labeling, the ^13^C_6_-CL pattern was rich in CL66:3, CL 66:4, CL68:4, and CL68:5, whereas unlabeled CL contained only trace amounts of those species and instead was rich in CL70:7, CL72:8, and CL72:9. Importantly, the transition from newly synthesized (labeled) CL to mature (unlabeled) CL was inhibited by inactivation of *Abhd18* (Fig. 4). The data demonstrate that ABHD18 alters the molecular composition of CL by species-selective degradation in TAZ-deficient flies.

## Discussion

The present data establish *Abhd18* as a functional homolog of *cld1*, the yeast lipase that provides substrates to TAZ by removing a fatty acid from CL. *Abhd18* inactivation (i) increased the abundance of CL, (ii) prevented the accumulation of CL degradation products, such as MLCL and DLCL, and (iii) decreased the turnover of CL, MLCL, and DLCL as measured by ^13^C incorporation. Together, the data demonstrate that ABHD18 breaks down CL by removing fatty acids. ABHD18 is conserved among vertebrates, insects, and other animals and has been described as the *cld1* homolog of humans in a recent patent (18). This marks ABHD18 as a potential target for novel therapeutic approaches to Barth syndrome. Barth syndrome is an X-linked disease caused by *Taz* mutations (7), resulting in loss of CL remodeling (19). Barth syndrome is associated with critically low levels of CL, directly caused by excessive CL degradation (20), which makes the inhibition of ABHD18 a plausible choice for drug development.

**Figure 5:**
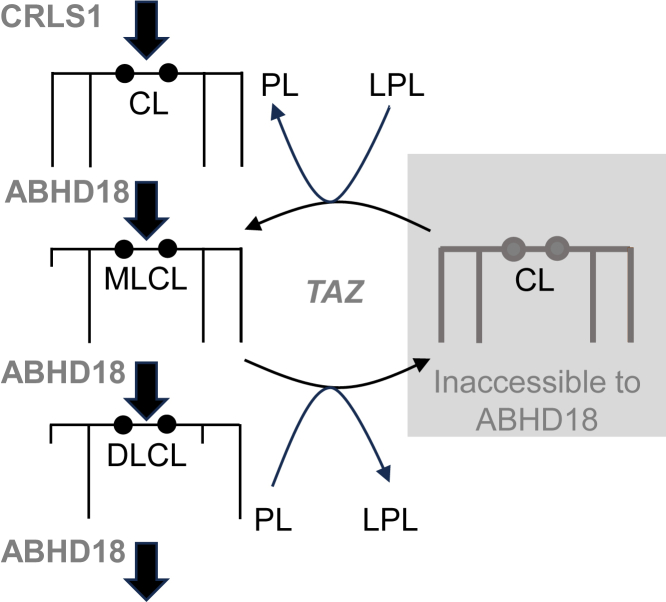
**Function of ABHD18 and TAZ in CL metabolism.** The *cartoon* depicts the proposed mechanism of CL degradation and remodeling. CRLS1 (CL synthase) forms CL, and ABHD18 removes CL by repeated deacylations. TAZ redistributes fatty acids between MLCL, CL, phospholipids (PLs), and lysophospholipids (LPLs). Remodeled CL is inaccessible to ABHD18. ABHD, α/β-hydrolase domain; CL, cardiolipin; MLCL, monolysocardiolipin; TAZ, tafazzin.

In addition, our results have important implications for the mechanism of CL remodeling. It is widely believed that repetitive cycles of hydrolysis and reacylation remodel the fatty acids of CL. If that were the case, inhibition of either hydrolysis or reacylation would decrease the turnover of the acyl chains attached to CL but have no effect on the turnover of the glycerol backbone. However, here we show that ABHD18 and TAZ do affect the turnover of the CL backbone, albeit in opposite ways, and previously, we have shown that Cld1 and Taz1 have opposite effects on the turnover of the acyl chains of CL (21). This is inconsistent with the orthodox remodeling mechanism but suggests that ABHD18 and TAZ have opposing effects on CL metabolism. We show that ABHD18 breaks down CL by stepwise deacylations, a function that is separate from CL remodeling. In contrast, TAZ prevents CL degradation by engaging MLCL in transacylations. Once MLCL is formed, TAZ alone is sufficient to remodel CL by repetitive forward and reverse reactions, transferring desired fatty acids from phospholipids to MLCL and undesired fatty acids from CL to lysophospholipids (6, 22). We hypothesize that remodeling is energetically driven by the high protein density in mitochondrial cristae, which provokes packing stress in the lipid bilayer, and that remodeled CL is trapped in protein complexes (4, 20, 21, 23). Thus, ABHD18 and TAZ operate on separate but interdependent pathways. ABHD18 catalyzes CL degradation, which is thwarted by TAZ, and TAZ catalyzes CL remodeling, which depends on ABHD18 to create a free hydroxyl group (Fig. 5).

Apart from its participation in TAZ-catalyzed remodeling, ABHD18 alters the CL species composition by species-selective degradation. The mechanism by which ABHD18 selects CL species for degradation cannot be inferred from the present data. Hypothetically, the enzyme may be specific for certain molecular species as has been reported for Cld1 (24, 25). Alternatively, protein-rich domains may sequester CL species selectively and make them inaccessible to ABHD18. CL species that were resistant to ABHD18 contained more double bonds and less methylene groups than susceptible CL species. Such features promote lipid–protein interactions, supporting the idea that protein association protects CL from degradation.

It is possible that lipases other than ABHD18 contribute to the deacylation of CL. Several members of the phospholipase A2 (PLA2) superfamily are able to attack CL *in vitro*, among them iPLA2, sPLA2, and cPLA2 (26). Among those, iPLA2s are present in mitochondria. Inactivation of iPLA2β reduced the accumulation of MLCL in ΔTAZ *Drosophila* (27) but to a much lesser extent than the inactivation of ABHD18. iPLA2γ, an enzyme listed in the high-confidence proteome of human mitochondria (14), was shown to cleave oxidized fatty acids from CL (28) and to be essential for bioenergetic function (29). Thus, iPLA2s may contribute to CL degradation in mitochondria, but in contrast to ABHD18, iPLA2s only remove a single fatty acid, forming MLCL (26).

In summary, we have shown that ABHD18 degrades CL by stepwise removal of its fatty acids. In the wildtype, the degradation pathway is largely suppressed. The underlying mechanism remains to be established, but we propose that TAZ-dependent remodeling sequesters CL in protein complexes, which makes CL inaccessible to ABHD18. In the absence of TAZ, CL becomes unprotected and is rapidly degraded by ABHD18, causing CL deficiency and mitochondrial dysfunction. In the absence of ABHD18, lack of the degrading enzyme causes CL to accumulate. Although the phenotype of ABHD18 deficiency may not be as severe as the phenotype of TAZ deficiency, ABHD18 has probably important biological function(s). CL, like any other molecule, must be eliminated from the cell in order to maintain metabolic equilibrium. We are thus hypothesizing that CL-degrading lipases, like Cld1 and ABHD18, are essential for the turnover of mitochondrial membranes and for mitochondrial quality control. This concept is supported by data in yeast, showing that Cld1 eliminates oxidized CL (24) and that *cld1* deletion shortens the chronological lifespan under stress conditions (10).

## Experimental procedures

### Myoblast cultures

C2C12 mouse myoblasts with and without *Taz*KO (30) were grown in Dulbecco's modified Eagle's medium (Gibco) containing 4.5 g/L glucose, supplemented with 10% fetal bovine serum, 2 mM glutamine (Gibco), penicillin (100 units/ml), and streptomycin (100 μg/ml), under standard conditions (5% CO_2_ at 37 °C). siRNAs targeted to *Abhd4*, *Abhd10*, *Abhd11*, and *Abhd18* (3 different 27mers for each gene) were purchased from OriGene. EndoFectin Max Transfection Reagent from GeneCopoeia was used for siRNA transfection of *Taz*KO C2C12 mouse myoblasts according to the manufacturer's instructions. Ninety-six hours post transfection, cells were lysed in TRI Reagent Solution from ThermoFisher Scientific, and total RNAs were isolated with Qiagen's RNeasy Mini Kit. To assess knockdown efficiency, total RNAs were quantified with a NanoDrop Microvolume Spectrophotometer (ThermoFisher Scientific), and equal amounts were then used to synthesize complementary DNA (cDNA) with a Verso cDNA Synthesis Kit from ThermoFisher Scientific. PowerUp SYBR Green Master Mix from ThermoFisher Scientific was used to perform quantitative real-time PCR on a StepOne Real-Time PCR System (ThermoFisher Scientific) according to the manufacturer's instruction.

### Drosophila

Fly strains were maintained on Formula 4-24 Instant Drosophila Medium from Carolina Biological Supply in 3-inch culture vials at 24 °C. The ΔTAZ strain used in this study is TAZ^889^, a new mutant allele generated in w1118 background by WellGenetics, which has the same lesion as our previously published allele (31), but with a red fluorescent protein marker knocked into the deleted region to facilitate introduction of additional transgenic elements (32). The wildtype strain used in this study is w1118. Δ^1^ABHD18 was purchased from Kyoto Drosophila Stock Center (Stock #: 140919, genotype: y∗ w∗; PBac{SAstopDsRed}LL05079 P{FRT(whs)}2A P{neoFRT}82B P{Car20y}96E/TM6B, Tb^1^). It has a transposon inserted into a coding exon common to all splice variants. Δ^2^ABHD18 was purchased from Bloomington Drosophila Stock Center (Stock #: 85387, genotype: w^1118^; PBac{PB}CG32112c07055/TM6B, Tb^1^). It has a different transposon inserted into a noncoding region (exon of one splice variant and intron of several other splice variants). To assess the effect of transposon insertions on *Abhd18* gene expression, total RNAs were isolated from the homozygous insertion strains and equal amounts were reverse-transcribed into cDNA, which were then quantified by real-time PCR with the forward primer 5′-AGCACAAGCCCATATGCATTC-3′ and the reverse primer 5′-GCCATAGAAAGGATTCTCCAT-3′. Male fertility was assessed by placing five 1-week-old males with five 1-week-old virgin w1118 females in a culture vial for 5 days at 24 °C followed by counting the F1 progeny 2 weeks later. Three vials were set up and scored for each genotype tested. Thoraces and abdomens were dissected under the microscope after immobilizing flies with carbon dioxide.

### Lipid analysis

Lipids were extracted into chloroform–methanol (33). Samples were homogenized in water, and the protein concentration was measured (34). Aliquots of the homogenates, corresponding to 0.5 to 1 mg protein, were suspended in methanol:chloroform (2:1) and incubated at 37 °C for 30 min to denature proteins. Internal standard, containing 1 nmol of CL15:0_15:0_15:0_16:1, was added. Chloroform and water were added, the samples were vortexed, and phase separation was achieved by centrifugation. The lower phase was collected, dried under nitrogen, and redissolved in 0.2 ml chloroform:methanol (1:1). Lipids were analyzed by LC–ESI–MS/MS on a QExactive HF-X instrument coupled directly to a Vanquish UHPLC (Thermo Fisher Scientific). An aliquot of 5 μl was injected into a Restek Ultra C18 reversed-phase column (Restek Corporation; 100 × 2.1 mm; particle size 3 μm) that was kept at a temperature of 50 °C. Chromatography was performed with solvents A and B at a flow rate of 0.15 ml/min. Solvent A contained 600 ml acetonitrile, 399 ml water, 1 ml formic acid, and 0.631 g ammonium formate. Solvent B contained 900 ml 2-propanol, 99 ml acetonitrile, 1 ml formic acid, and 0.631 g ammonium formate. The chromatographic run time was 40 min, changing the proportion of solvent B in a nonlinear gradient from 30 to 35% (0–2 min), from 35 to 67% (2–5 min), from 67 to 83% (5–8 min), from 83 to 91% (8–11 min), from 91 to 95% (11–14 min), from 95 to 97% (14–17 min), from 97 to 98% (17–20 min), from 98 to 100% (20–25 min), and from 100 to 30% (25–26 min). For the remainder of the run time, the proportion of solvent B stayed at 30% (26–40 min). The mass spectrometer was operated in negative ion mode. The spray voltage was set to 4 kV, and the capillary temperature was set to 350 °C. MS1 scans were acquired in profile mode at a resolution of 120,000, an automatic gain control target of 1e6, a maximal injection time of 65 ms, and a scan range of 200 to 2000 *m/z*. MS2 scans were acquired in profile mode at a resolution of 30,000, an automatic gain control target of 3e6, a maximal injection time of 75 ms, a loop count of 7, and an isolation window of 1.7 *m/z*. The normalized collision energy was set to 30, and the dynamic exclusion time was set to 31 s. For lipid identification, data were analyzed by the software LipidSearch 5.1.8 (Thermo Fisher Scientific). The general database of LipidSearch was searched with a precursor tolerance of 2 ppm, a product tolerance of 0.01 Da, and an intensity threshold of 1.0%. Molecular ions of CL, MLCL, and DLCL were included in the analysis only if they were associated with confirmatory fragmentation spectra (grades A and B). To calculate MLCL/CL ratios, the sum of the intensities of all MLCL species was divided by the sum of the intensities of all CL species.

### ^13^C isotopomer analysis

Flies were kept on a labeling medium consisting of five parts of ^13^C_6_-glucose (Cambridge Isotope Lab, Inc) and one part of yeast paste (by weight). The medium was mixed in a small amount of water and placed on a square of filter paper (0.25 × 0.25 inch). After 48 h, thoraces and abdomens were dissected, and lipids were extracted and analyzed by mass spectrometry as described previously. Mass spectra were imported in the Quality Browser of Xcalibur (version 4.1.50). The intensities of ^13^C_0_, ^13^C_3_, ^13^C_6_, and ^13^C_9_ isotopomers of CL species were averaged over 0.2-min intervals centered at the peak retention time of the respective species (16).

## Data availability

All data are contained within the article. Raw data of mass spectra are available upon request (michael.schlame@med.nyu.edu).

## Supporting information

This article contains supporting information.

## Conflict of interest

The authors declare that they have no conflicts of interest with the contents of this article.
