# Supplementary material for: ABHD18 degrades cardiolipin by stepwise hydrolysis of fatty acids
Source: J Biol Chem. 2025 May 14;301(6):110237. doi: 10.1016/j.jbc.2025.110237 (PMC12173737; doi:10.1016/j.jbc.2025.110237)

Supporting Information

ABHD18 degrades cardiolipin by stepwise hydrolysis of fatty acids

Mindong Ren, Shiyu Chen, Miriam L. Greenberg, Michael Schlame

Table S1, Figure S1

Table S1. Probabilities of mitochondrial localization of ABHD proteins. The software DeepLoc and MULocDeep were used to predict the most likely intracellular compartment and the probability of mitochondrial localization. Only ABHD10, 11, and 18 were predicted to be localized in mitochondria by both programs.

Protein	Probability of mitochondrial localization		Predicted intracellular compartment	
	DeepLoc	MULocDeep	DeepLoc	MULocDeep
ABHD1	0.3931	0.1388	ER	Cell membrane
ABHD2	0.4654	0.0389	ER	Cell membrane
ABHD3	0.3624	0.0612	ER	Cell membrane
ABHD4	0.5159	0.0128	Lysosome	Cytoplasm
ABHD5	0.364	0.0196	ER	Cytoplasm
ABHD6	0.3558	0.8008	ER	Mitochondria
ABHD7	0.4733	0.01	Golgi	Golgi
ABHD8	0.2638	0.0077	Cytoplasm	Cytoplasm
ABHD9	0.3592	0.0096	ER	ER
**ABHD10**	**0.8732**	**0.8349**	**Mitochondria**	**Mitochondria**
**ABHD11**	**0.9122**	**0.8175**	**Mitochondria**	**Mitochondria**
ABHD12	0.2744	0.1591	ER	Er
ADBH12B	0.4595	0.0454	ER	Cytoplasm
ADBH13	0.3554	0.0299	ER	ER
ABHD14A	0.059	0.0102	ER	Secreted
ABHD14B	0.1399	0.0241	Cytoplasm	Cytoplasm
ABHD15	0.4334	0.0125	ER	ER
ABHD16A	0.5041	0.72	ER	Mitochondria
ABHD16B	0.7419	0.074	ER	Cytoplasm
ABHD17A	0.1839	0.0033	Lysosome	Cell membrane
ABHD17B	0.1733	0.003	Lysosome	Cell membrane
ABHD17C	0.2153	0.0148	Lysosome	Cell membrane
**ABHD18**	**0.7936**	**0.48**	**Mitochondria**	**Mitochondria**

Figure S1, A. Superimposed AlphaFold structures of CLD1 (light pink) and ABHD18 (beige). AlphaFold structures of CLD1 and mouse ABHD18 were downloaded from UNIPROT and imported into ChimeraX. The Matchmaker Function was used to superimpose the structures onto each other. The catalytic triads (serine-histidine-aspartate) have nearly identical spatial relations.

Figure S1, B. Docking of CL to the active sites of CLD1 and ABHD18. Docking of CL was done in AutoDockVina. The structure of tetrastearoyl-CL ([3-[[(2~{R})-2,3-di(octadecanoyloxy)propoxy]-oxidanidyl-phosphoryl]oxy-2-oxidanyl-propyl] [(2~{R})-2,3-di(octadecanoyloxy)propyl] phosphate) was obtained from the RCSB Protein Databank. A search space of 50x50x50 Angstroms was chosen, which included the catalytic triad. Ten different docking orientations were produced, of which one was selected. In this orientation, an ester bond was close to the catalytic serine and histidine residues. The catalytic triads (D392, H424, S230 for CLD1 and D409, H436, S199 for ABHD18) are shown in the same colors as in panel A.

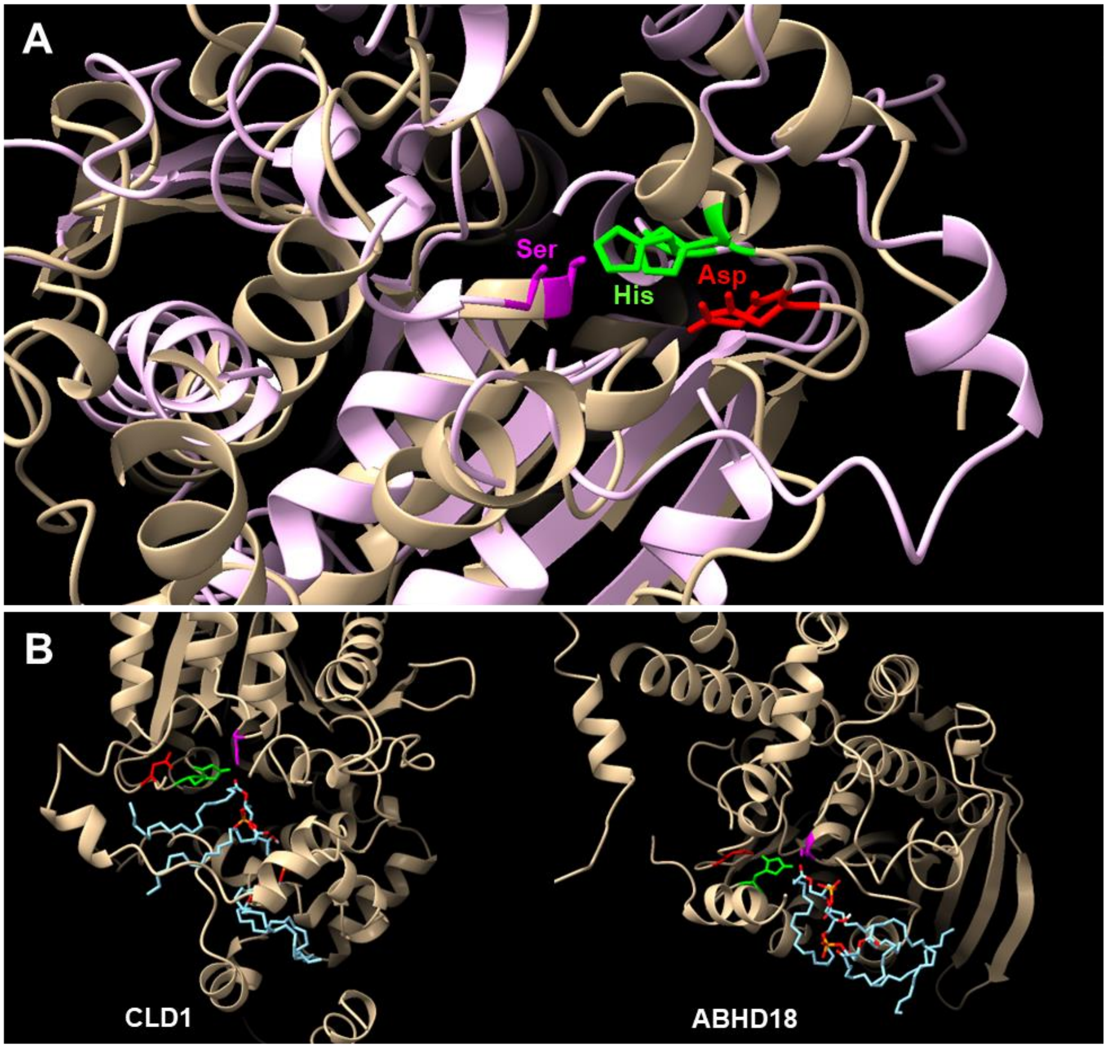

Supplement: Supplemental Table S1 and Fig. S1 [file mmc1.pdf]
